# Intolerance of loud sounds in childhood: Is there an intergenerational association with grandmaternal smoking in pregnancy?

**DOI:** 10.1371/journal.pone.0229323

**Published:** 2020-02-24

**Authors:** Amanda Hall, Kate Northstone, Yasmin Iles-Caven, Genette Ellis, Steve Gregory, Jean Golding, Marcus Pembrey

**Affiliations:** 1 School of Life and Health Sciences, Aston University, Birmingham, England, United Kingdom; 2 Bristol Medical School (Public Health Sciences), University of Bristol, Bristol, England, United Kingdom; 3 Centre for Academic Child Health, Bristol Medical School (Public Health Sciences), University of Bristol, Bristol, England, United Kingdom; University of Wyoming College of Health Sciences, UNITED STATES

## Abstract

Recent research using the Avon Longitudinal Study of Parents and Children (ALSPAC) demonstrated an association between maternal grandmother smoking in pregnancy and the autistic traits of impaired social communication and repetitive behaviour in granddaughters but not grandsons, but of paternal grandmother smoking and early development of myopia in the grandchild. Here we investigate whether grandmaternal smoking in pregnancy is associated with intolerance to loud sounds. ALSPAC collected information during the index pregnancy from the study parents on the smoking habits, social and other features of their own parents. Maternal report when the child was aged 6 and 13 included hating loud sounds; at age 11 the child was tested for volume preference for listening to music through headphones. Statistical analysis compared results for grandchildren in relation to whether a parent had been exposed in utero to maternal smoking, adjusted for their grandparents’ social and demographic attributes. We hypothesised that there would be sex differences in the effects of grandmaternal prenatal smoking, based on previous intergenerational studies. For 6-year-old children maternal report of intolerance to loud noise was more likely in grandsons if the maternal grandmother had smoked [adjusted odds ratio (AOR) 1.27; 95% confidence interval (CI) 1.03,1.56; P = 0.025], but less likely in girls [AOR 0.82; 95%CI 0.63,1.07] P_interaction_ <0.05. If the paternal grandmother had smoked the grandchildren were less likely to be intolerant, especially girls. The objective measure of choice of volume for music through headphones showed that grandsons of both maternal and paternal smoking grandmothers were less likely to choose high volumes compared with granddaughters (P<0.05). In line with our prior hypothesis of sex differences, we showed that grandsons were more intolerant of loud sounds than granddaughters particularly at age 6, and this was confirmed by objective measures at age 11.

## Background

The Avon Longitudinal Study of Parents and Children (ALSPAC) birth cohort was established in 1991 to study developmental phenotypic variation across the local population in relation to inherited DNA variants and a wide range of dietary, social and environmental exposures. **[1]** Maternal exposures during and before the study pregnancy was one interest during the first decade, being relevant to the Developmental Origins of Health and Disease (DOHaD) hypothesis **[2,3]** and the emergence of environmental epigenetics **[4,5]**. By the second decade, ALSPAC began exploring associations with grandmaternal exposures in the light of experimental evidence of mammalian epigenetic inheritance across several generations (see below). Whilst population-wide traits have been the primary ALSPAC outcome measures, doctor-diagnoses are recorded and, in common multifactorial conditions, the two may be considered together. Two recent examples of this are postnatal refractive eye development focusing on refractive errors underlying myopia (near sightedness) **[6]** and four childhood behavioural traits predictive of autism spectrum disorders (ASD) and diagnosed ASD **[7]**.

Since the launch of human genome wide association studies (GWAS) in 2007 **[8]** the mainstream view of the inheritance of complex traits and multifactorial diseases is that the heritability will eventually be explained by the sum total of common and increasingly rare DNA sequence variations inherited by the individual, plus ‘de novo’ mutations arising early in development. As whole genome sequencing takes off, there is some support for this view with respect to the genetics of height **[9]**, no support from studies of Type 2 diabetes **[10,11]** and a speculative reappraisal of what complex traits actually are **[12]**. In parallel, the last decade has seen mammalian evidence of non-DNA-sequence-based inheritance induced by parental/ancestral experiences **[13]**. These effects are called *intergenerational* if the exposure (or importantly the organism’s response to it, e.g. response to DNA damage) could have reached the germ cells leading to the next generation(s), or *transgenerational* if this is not the case. The latter implies some molecular ‘memory’ of the ancestral experience, or its response to it, is being passed down via the gametes. We have written ***inter/trans-generational*** when both apply.

The molecular basis of intergenerational or transgenerational epigenetic inheritance is poorly understood, particularly in humans, but it does include altered DNA methylation, histone modification and exposure induced non-coding RNAs being transferred to sperm **[14,15]**. Thus, what has, in the past, been interpreted as ‘likely to be genetic’, is now better considered more broadly ‘as likely to be factors inherited at conception’**[16]**. In line with this view, ALSPAC has included assessment of associations between parental/ancestral early-life experiences and developmental variation in the study participants. Unlike initial human epidemiological studies by others of associations with parental/ancestral exposure to historic famines **[17,18]** we selected cigarette smoking in the early 2000’s as a suitable exposure for proof of principle studies in a contemporary population.

As described in detail elsewhere **[19]** the intention at the start was to see if we could replicate some of the distinctive features of human intergenerational and transgenerational associations found by others, principally the exposure sensitive period(s) during development. We found support for the mid-childhood sensitive period, initially shown by Bygren and colleagues **[18]**, and noted a sex-specific bias in the intergenerational association with paternal onset of regular smoking before 11years. This led to a collaboration with Professor Bygren and colleagues to test for sex-specific effects in the Swedish data; and striking sex-specific (but not sex-limited) associations were found **[20]**. In view of this, we started a programme on intergenerational association studies within ALSPAC with smoking as the exposure of interest. We began by analysing the child’s growth and anthropomorphic measures in relation to study fathers who started regular smoking before puberty (<11ys) **[21]** and with either grandmother smoking in pregnancy **[22]**. We have since extended these intergenerational studies to behavioural and sensory perception measures collected prospectively in the study participants.

The hypersensitivity measures were intolerance of loud sounds and the ability to detect bitter taste using 6-*n*-propylthiouracil (PROP) **[23]**. Here we consider intolerance of loud sounds in relation to grandmaternal smoking. Aetiological studies of intolerance to loud sounds *per se* have rarely been undertaken but may be incorporated into studies of hyperacusis; namely intolerance to everyday or low intensity sounds. Hyperacusis is a feature of certain monogenic disorders, such as Williams syndrome **[24]** and Smith-Megenis syndrome **[25]** and occurs in about 20% of children with the multifactorial disorder ASD **[26,27]**. In adults with hyperacusis who are otherwise neurotypical, the medial olivocochlear (MOC) efferent reflex is stronger than usual, as it is in children with ASD and coexisting hyperacusis **[28]**. Among the candidate genes for ASD, the contactin genes CNTN5 and CNTN6 code for neural cell adhesion proteins that promote neurite outgrowth and synaptogenesis.**[29]**. CNTN6 mutations, in particular, are risk factors for abnormal auditory sensory perception amongst those with ASD **[30]**. Our earlier finding of an association between maternal grandmother smoking in pregnancy and impaired social communication and repetitive behaviour in granddaughters but not grandsons **[7]** provides an example of intergenerational responses to maternal grandmaternal smoking in pregnancy. However, the present study of intolerance to loud sounds was not intended or designed to focus on ASD.

There is a substantial experimental animal literature on the ways in which specific environmental exposures can influence the phenotype of subsequent (unexposed) generations through non-DNA sequence-based inheritance **[13,31,32]**. In relevant animal experiments exposures are often delivered to a pregnant dam thereby exposing her offspring throughout fetal life. The dam is traditionally labelled F0, so we have adopted the same notation for the study grandmothers, F1 for their offspring–the study parents, and F2 for the study participants. The results of animal experiments support the human epidemiological observations and provides numerous examples of sex-specificity or sex-bias in both the route of transmission down the generations and the phenotypic outcomes in descendants. As mentioned above, sex-specificity was a feature of initial human studies and this is true of early rodent experiments **[33,34,35]**.

The transmissions (progressing from intergenerational to transgenerational) can be complicated as illustrated by the study of maternal high-fat diet exposure in mice **[36]**. This resulted in an increase in body size and reduced insulin sensitivity in both sexes that persisted across two generations (F1 & F2) via both maternal and paternal lineages. However, at the next generation (F3) only the females had increased body size and this effect was only passed on via the paternal lineage. Another example is a study that induced chronic social instability in adolescent mice (F0) and then followed their unexposed descendants (F1, F2 & F3) **[37]**. Only female descendants exhibited anxiety-like behaviour and social deficits, but after F1 this phenotype is only transmitted through males who themselves do not manifest anxiety-like behaviour and social deficits. This latter study was the starting point for a commentary that explored why sex matters in such intergenerational and transgenerational responses.**[38]** It could not provide any conclusive explanations for the sex-specificity but recognised that the phenomenon was common. It is important to emphasise that intergenerational phenotypic responses can be widespread, for example combine metabolic and behavioural features **[39]** and include beneficial outcomes **[40]**.

There is not necessarily transmission of the initial phenotype, although inheritance of an acquired characteristics can occur. Sometimes the F1 or F2 phenotypic can change to be in the opposite direction, as if the response is a corrective adaptation **[41]**. Increasingly animal experiments permit organ/cell specific gene expression and epigenomic studies. These can include germline, sperm, ovum or early embryo analyses to elucidate the molecular mechanisms that mediate intergenerational transmissions **[31,32,42]**. Such opportunities are limited in humans, although there have been ingenious studies to elucidate the nature of the epigenome in the conceptus and its trajectory into the human pre-implantation embryo **[43]**.

Overall human results from epidemiological, observational studies over the last two decades have been in line with rodent experiments (reviewed in **[44]**). The inter/trans-generational responses can be through either the paternal or maternal line; and like rodent experiments, human observations often demonstrated a sex-specificity (but not necessarily sex limitation) in either the parental route of transmission and/or the offspring phenotypic outcomes. This is true of most of the human studies referenced below since 2005 and why we have hypothesised that there will be sex-specific differences in any transgenerational associations detected in this study of intolerance to loud sounds. Human studies have defined ‘exposure-sensitive periods’ during early development; namely fetal life and mid-childhood just before puberty. These exposure times are broadly supported by responses to swings in F0 food supply **[17,19,33,45,46]** or F0 smoking **[21,47,48, 49]**. However, some F0 pre-conceptional exposures in (early) adulthood can also be associated with phenotypic changes in the next generation **[21]**.

Exposure to famine is likely to be confounded by psychological stress, and F0 exposure to stress *per se* has also been reported to be associated with altered psychological state or early development in the next generation **[50,51]**. As described above, smoking was selected in the early 2000’s as an exposure (with available data) in a contemporary population with which to valid the exposure sensitive periods of fetal life and mid-childhood as indicated by studies of historic famines. Smoking can clearly initiate intergenerational responses, which raises the question as to how it can induce the relevant molecular change(s) in the gametes or the resulting conceptus. It is important to note that tobacco smoking causes DNA damage **[52]**. This damage or the body’s response to it **[53]** may well be the key ‘signal’ that is transmitted to the next generation(s); and with such a generic genomic stress, the impact on development may be widespread.

## Material and methods

### The study subjects

The Avon Longitudinal Study of Parents and Children (ALSPAC) is an ongoing study which started during 1990–1992 by attempting to enrol all pregnant women resident in a defined area of Avon (in south-west England) with expected date of delivery between 1^st^ April 1991 and 31^st^ December 1992. Avon comprises the city of Bristol, surrounding urban and semi-urban areas, as well as rural communities. It has a total population of about 1 million people. A variety of methods were used to encourage enrolment in the study, using the health services, local publicity, contacts through chemists (drug stores), libraries and mother and toddler groups **[54]**. Approximately 80% of the eligible population took part in the study. The initial number of pregnancies enrolled is 14,541 (for these at least one questionnaire had been returned or a “Children in Focus” clinic had been attended by the 19^th^ July 1999). Of these initial pregnancies, there was a total of 14,676 foetuses, resulting in 14,062 live births and 13,988 children who were alive at 1 year of age. Comparison of the population enrolled with the census records of families with babies resident in the area in 1991 showed that the ALSPAC population was fairly representative although there was a slight bias in that those from the less well educated group were under-represented **[54,55,56]**.

Data were collected using a variety of methods, the most relevant to this research were: (a) self-completion questionnaires to the parents, answered in their own homes and returned to the study centre by post; (b) annual hands-on examinations of the children from the age of 7; (c) blood collected for genetic and epigenetic studies at different time points. The study website contains details of all the data that is available through a fully searchable data dictionary and variable search tool: http://www.bristol.ac.uk/alspac/researchers/our-data/. Data are available to bona fide applicants provided that by doing so no ethical or confidentiality guidelines are broken.

Ethical approval for the study was obtained from the ALSPAC Ethics and Law Committee [ALEC; IRB00003312] (registered on the Office of Human Research Protections database as U Bristol IRB #1). ALEC agreed that consent was implied if questionnaires were returned. Informed written consent was obtained for all biological samples prior to analysis, and for certain invasive procedures during the hands-on assessments (which were optional to attend) from the participant and/or legal guardian **[57]**. All study methods were performed in accordance with relevant guidelines and regulations. Together with the local Health Services ethics committees ALEC has approved the linkage of the DNA and methylation data to the detailed assessments and other information on the parents and children. Analyses of biological samples including genetic and DNA methylation are only carried out for individuals for whom informed generic consent has been received. Further detailed information on the ways in which confidentiality of the cohort is maintained may be found on the study website: http://www.bristol.ac.uk/alspac/researchers/research-ethics/

### The auditory outcome variables

Intolerance to loud sound was determined through parental questionnaire. When the child was 81 months (6 years) old, the mother was sent a questionnaire which included the question: ‘*Does he/she prefer music or talking to be loud or soft*?*’* with possible answers *(i) She hates loud sounds; (ii) He/She doesn’t mind if it’s loud or not; (iii) He/She loves loud sounds; (iv) Can’t say*. In all 8391 answers were received (an additional 124 were not completed). The frequency of answers was: (i) 10.9%; (ii) 73.7%; (iii) 11.4% and (iv) 3.9%. For this study we have compared those who hated loud sounds (10.9%) with all other children (89.1%).

At the age of 157 months (13 years), an identical question was asked of the mother in regard to her child. There were 7094 valid responses and an additional 71 where the question had been left blank. The frequency of answers was (i) 5.4%; (ii) 72.7%; (iii) 17.2% and (iv) 4.7%. The analyses in this paper compare those who hated loud sounds (5.4%) with the remainder (94.6%).

As a validation analysis we used an objective measure of the child’s comfortable loudness listening level measured when the children were age 11. Those who attended the hearing session of the Focus 11 clinic, were played a short piece of music through a portable CD player and supra-aural, Sennheiser PX40 headphones. They were asked to set the CD player at the volume level at which they would typically listen to music. The music was composed specifically for the purpose and thus had not previously been heard by the children. The sound pressure level of the CD player and headphones was calibrated using a KEMAR manikin (head and torso simulator) to give the equivalent sound pressure level at the eardrum for each volume setting. Each increment on the volume setting was equivalent to a change of 0.9–1.2 dB.

The volume level set by the child was noted. We identified the binary variable of the highest 10% of scores as a measure of tolerance to loud sounds, with the expectation that those who are intolerant to loud sounds would choose a lower volume level on the headphone test. The top 10% were listening on average at volume levels between 79 and 85 dB (A), where 85 dB (A) is equivalent to the sound of a noisy restaurant or food blender. The UK Control of Noise at Work Regulations (2005) state that exposure to noise of 85 dB (A) or above in the workplace (daily or weekly average exposure) is the level of sound at which employers must provide ear protection for their workers **[58]**. Consequently, we expected those who declared that they hated loud sounds to be under-represented in this group. A direct comparison showed that the mean volume level chosen by 388 11-year-olds who were said to hate loud noises at age 6 was significantly lower than that of the 3113 who did not hate such noises (mean difference -0.538; SE 0.314; P <0.05). A comparison of the 182 who were said at age 13 to hate loud noises, with the remaining 3295 showed a larger mean difference (-2.912; SE 0.445; P<0.0001), equivalent to around 3 dB quieter, thus indicating that the listening level measure was more closely aligned with the 13-year-old outcome.

Hearing function was assessed at age 11. Air conduction hearing thresholds were measured at 0.5, 1, 2 and 4 kHz, in both the left and right ears using a GSI 61 audiometer in a sound treated booth. Average hearing thresholds across 0.5–4 kHz were calculated for both ears. Ipsilateral acoustic reflexes were measured using a GSI 38 tympanometer: on completion of tympanometry the presence of ipsilaterally evoked acoustic reflexes were measured at 85, 95, and 105 dB. The left ear was tested first and the lowest level sound at which the reflex was evoked was recorded. If no reflex was present it was recorded as absent.

### The exposures

Within one of the questionnaires that was sent to each study parent (F1) during pregnancy, was a question as to whether their own mothers (F0) had smoked, and if so whether they had smoked during the pregnancy that resulted in their own birth. Where the parent knew that their mother had smoked but had not known whether she had smoked during the pregnancy, we assumed that she had done so. We have shown elsewhere, by looking at the study mother’s own birthweight that this was a valid assumption since the mean birthweight of the mothers who had been exposed as fetuses was 148g less than that of the women born to women we have assumed not to have smoked in pregnancy **[14]**. Using this definition of grandmother (F0) smoking in pregnancy results in a prevalence of fetal exposure for our study mothers and fathers (F1) of 37.9% and 42.1% respectively.

### Potential confounders

We examined the backgrounds of each of the grandparents (F0) in regard to each of the outcomes listed above. We compared their years of birth, their ages at the birth of their offspring (F1), their ethnic background (white; non-white), education level (the equivalent of ≥O-level; < O-level); their ages at the birth of the parent; the grandmother’s parity at the birth of the study parent (no previous children; previous children); the social class based on their occupations, categorised using the UK standard classification system **[59]**; whether they were smokers. We selected the variables identified as significantly associated with each specific outcome indicating reaction to loud sounds to be potential confounders.

### Statistical analyses

Initial analyses compared the unadjusted risk of each grandchild (F2) outcome with each of the parental fetal exposures. This was repeated, selecting whether the mothers themselves were smokers or non-smokers, and whether the grandchild was a boy or girl. Data were displayed as odds ratios with 95% confidence intervals.

For statistical adjustments, comparison was made of the risk of each potential confounder with each outcome, and those variables that had a P-value of < 0.10 were selected. These variables were then included in the logistic regression analyses according to whether the exposures were associated with the mother’s or father’s line. The variable concerning the relevant prenatal smoking of the grandmother was then incorporated into each analysis. The process was repeated according to whether the mother (F1) herself smoked or not, and for the sex of the grandchildren (F2). (We used the cut-point of P<0.10 in order not to omit any interesting or important associations throughout the paper).

## Results

### Responses

The proportion of study pregnancies that resulted in a child for whom the questions on loud sound intolerance were answered at ages 6 and 13 are shown in S1 Table, together with the proportion who attended the clinic at age 11 and performed the listening test. This shows a small difference in the likelihood of there being information available on the child (F2) according to whether the grandmother (F0) had smoked in pregnancy or not (e.g. for 6-year-old outcomes information was available for 57.4% of children whose maternal grandmothers had smoked prenatally v. 60.8% of children whose grandmother had not smoked in pregnancy). The same pattern was apparent for paternal grandmothers smoking in pregnancy and for each outcome–the information being slightly more likely to be available if the grandmother had not smoked.

### Maternal grandmother smoking and child intolerance to loud sound

The variation in unadjusted odds ratios for intolerance to loud sounds for all children showed little sign of any association with maternal grandmother smoking (Table 1). However, there was an increase in odds among the boys at age 6, especially when the mother did not smoke (P<0.10). Although the odds ratios for the girls were no different than would be expected by chance, the difference in odds between boys and girls showed a significant interaction (P < 0.05). There were no associations with intolerance to loud sounds at age 13.

**Table 1:** Unadjusted risk of the child hating loud sounds at ages 6 and 13 if the maternal grandmother had smoked in pregnancy. [P values <0.10 are in bold].

HATES LOUD SOUNDS	AT AGE 6			AT AGE 13		
	N	UOR [95%CI]	P	N	UOR [95% CI]	P
All children	7562	1.03 [0.89,1.20]	0.674	6087	0.93 [0.74,1.17]	0.559
Boys	3870	1.20 [0.99,1.46]	**0.062**	3034	0.92 [0.68,1.26]	0.619
Girls	3692	0.82 [0.65,1.04]	0.106	3053	0.95 [0.67,1.33]	0.748
		*				
Mother non-smoker						
All children	6355	1.03 [0.87,1.21]	0.749	5221	1.00 [0.78,1.28]	1.000
Boys	3242	1.21 [0.98,1.51]	**0.076**	2597	0.96 [0.69,1.34]	0.824
Girls	3113	0.81 [0.62,1.05]	0.118	2624	1.05 [0.73,1.50]	0.805
		*				
Mother smoker						
All children	1184	1.13 [0.78,1.64]	0.515	847	0.77 [0.38,1.55]	0.459
Boys	619	1.20 [0.75,1.92]	0.438	431	0.86 [0.36,2.04]	0.736
Girls	565	0.95 [0.51,1.78]	0.868	416	0.60 [0.17, 2.07]	0.417

*Significant difference between results for boys and girls; UOR = unadjusted odds ratio

Examination of the variables that were associated with intolerance to loud noises at 6 (Table 2 and S2 Table) identified the year of birth of each maternal grandparent (F0), the age of her father (F0) at the time of birth of the study mother (F1), and the ethnic background of each of her parents (F0) as potential confounders.

**Table 2:** Proportion (n) of children who hated loud noises at age 6 according to features of their grandparents. [P values <0.10 are in bold].

Variable	MGM	MGF	PGM	PGF
Year of birth				
Pre 1925	14.3% (108)	14.1% (179)	14.6% (114)	13.8% (164)
1925–1929	11.6% (115)	10.0% (133)	11.7% (89)	9.5% (81)
1930–1934	10.7% (166)	11.7% (184)	10.1% (101)	10.4% (95)
1935–1939	12.0% (208)	9.4% (136)	10.4% (93)	9.5% (70)
1940–1944	8.5% (119)	9.3% (91)	9.0% (59)	8.1% (33)
1945+	9.2%(96)	8.1% (42)	8.3% (26)	9.9% (14)
P	**<0.001**	**<0.0001**	**<0.001**	**<0.001**
N	7466	7014	4406	4239
Ethnic background				
White	10.8% (861)	10.8% (855)	10.7% (680)	10.7% (675)
Non-white	17.1% (21)	16.8% (24)	17.1% (21)	16.3% (24)
P	**0.030**	**0.025**	**0.026**	**0.032**
N	8069	8044	6470	6453
Education level				
Lower	10.3% (400)	10.5% (367)	10.4% (338)	11.3% (334)
Higher	11.5% (260)	10.8% (250)	11.8% (203)	10.8% (212)
P	0.142	0.716	0.111	0.760
N	6127	5788	4979	4986
Ever smoked				
Yes	11.1% (483)	11.0% (646)	10.3% (372)	10.9% (511)
No	10.6% (377)	10.7% (207)	11.4% (325)	11.3% (146)
P	0.402	0.697	0.162	0.684
N	7904	7803	6440	5989
Age at birth of parent				
<25 years	10.4% (286)	9.6% (131)	9.1% (169)	8.6% (81)
25–34	10.8% (415)	10.9% (443)	11.5% (337)	10.4% (314)
35+	12.6% (111)	12.1% (191)	11.4% (84)	12.5% (169)
P	0.121	**0.046**	**0.022**	**0.003**
N	7466	7014	5527	5308
Parity				
0	10.5% (273)	-	10.4% (99)	-
1+	11.1% (619)		11.9% (187)	
P	0.396		0.242	
N	8171		2528	
Smoked prenatally				
Yes	11.0% (303)	-	9.7% (253)	-
No	10.8% (552)		11.6% (443)	
P	0.827		**0.018**	
N	8174		6412	
Social class				
P	0.365	0.838	0.270	0.188
N	4469	6636	3422	5978

Once these were taken into account (Table 3), the associations for boys at age 6 became significant at P<0.05, and there were still significant interactions between the sexes with the girls being less likely to be intolerant to loud noises and the boys more so. This was true for all children as well as for those whose mothers did not smoke. In contrast there were no such associations for the children who were intolerant to loud noises at age 13.

**Table 3:** Adjusted risk of the child hating loud sounds at ages 6 and 13 if the maternal grandmother had smoked in pregnancy. [P values <0.10 are in bold]. Adjusted for year of birth of each grandparent, age of grandfather at birth of the mother, ethnic origins of both grandparents.

HATES LOUD SOUNDS	AT AGE 6			AT AGE 13		
	N	AOR [95%CI]	P	N	AOR [95% CI]	P
All children	6657	1.07 [0.91,1.26]	0.413	5467	1.02 [0.98,1.05]	0.313
Boys	3436	1.27 [1.03,1.56]	**0.025**	2752	0.96 [0.69,1.33]	0.797
Girls	3221	0.82 [0.63,1.07]	0.143	2715	0.93 [0.64,1.35]	0.703
		*				
Mother non-smoker						
All children	5729	1.05 [0.88,1.26]	0.579	4769	0.98 [0.75,1.27]	0.870
Boys	2940	1.26 [1.00,1.59]	**0.046**	2389	0.98 [0.68,1.40]	0.906
Girls	2789	0.80 [0.59,1.07]	0.128	2380	0.98 [0.66,1.45]	0.916
		*				
Mother smoker						
All children	909	1.18 [0.77,1.82]	0.447	683	1.03 [0.48,2.19]	0.945
Boys	488	1.37 [0.80,2.34]	0.258	360	0.91 [0.36,1.27]	0.838
Girls	411	0.85 [0.41,1.77]	0.665	323	1.29 [0.32,5.14]	0.719

*Significant difference between results for boys and girls; AOR = unadjusted odds ratio

### Paternal grandmother smoking and child intolerance to loud sounds

The patterns were quite different if the paternal grandmother had smoked in pregnancy (Table 4). The unadjusted data for intolerance to loud noises at age 6 showed a reduced risk for all children and for girls, as well as for the children whose mothers did not smoke. Again, there were significant differences between the sexes, with the girls being less likely than the boys to be intolerant of loud noises (P<0.05). At age 13, all children were significantly more likely to have a reduced risk, with boys and girls having similar odds ratios. This was true of those whose mothers did not smoke, but when the mother did smoke, there was a significant difference between the sexes–the girls being more likely than the boys to be intolerant to loud noises.

**Table 4:** Unadjusted risk of the child hating loud sounds at ages 6 and 13 if the paternal grandmother had smoked in pregnancy. [P values <0.10 are in bold].

HATES LOUD SOUNDS	AT AGE 6			AT AGE 13		
	N	UOR [95%CI]	P	N	UOR [95% CI]	P
All children	6166	0.82 [0.69,0.96]	**0.015**	5032	0.72 [0.56,0.93]	**0.011**
Boys	3133	0.91 [0.73,1.12]	0.370	2501	0.72 [0.51,1.01]	**0.060**
Girls	3033	0.72 [0.56,0.92]	**0.010**	2531	0.73 [0.51,1.05]	**0.094**
		*				
Mother non-smoker						
All children	5289	0.79 [0.67,0.95]	**0.011**	4399	0.73 [0.56,0.95]	**0.019**
Boys	2685	0.93 [0.74,1.17]	0.523	2185	0.78 [0.55,1.12]	0.180
Girls	2604	0.65 [0.49,0.86]	**0.002**	2214	0.68 [0.47,1.00]	**0.051**
		*				
Mother smokes						
All children	857	1.01 [0.64,1.58]	0.976	615	0.81 [0.35,1.87]	0.616
Boys	441	0.81 [0.45,1.48]	0.501	309	0.32 [0.09,1.17]	**0.084**
Girls	416	1.40 [0.69,2.85]	0.355	306	3.29 [0.67,16.12]	0.141
					*	

*Significant difference between results for boys and girls; UOR = unadjusted odds ratio

Examination of the variables related to paternal grandparents that were associated with intolerance to loud noises at 6 and 13 (Table 2 and S2 Table) identified the year of birth of each of the grandparents (F0), the age of the grandparents (F0) at the time of birth of the study father (F1), and the ethnic background of each of his parents (F0) as potential confounders. The adjusted odds for children intolerant to loud noises were still reduced for all children but there were no longer interactions between the sexes with the exception of the group where the study mother (F1) had smoked: as in the unadjusted data at age 13, the girls were more likely than the boys to be intolerant to loud sounds (Table 5).

**Table 5:** Adjusted risk of the child hating loud sounds at ages 6 and 13 if the paternal grandmother had smoked in pregnancy. [P values <0.10 are in bold]. Adjusted for year of birth of each paternal grandparent, age of grandfather at birth of the father, ethnic origins of both grandparents.

HATES LOUD SOUNDS	AT AGE 6			AT AGE 13		
	N	AOR [95%CI]	P	N	AOR [95% CI]	P
All children	4075	0.81 [0.65,0.99]	**0.045**	3523	0.79 [0.59,1.06]	0.112
Boys	2086	0.83 [0.63,1.09]	0.177	1762	0.89 [0.60,1.33]	0.568
Girls	1989	0.78 [0.56,1.08]	0.135	1761	0.68 [0.44,1.06]	**0.090**
Mother non-smoker						
All children	3603	0.80 [0.64,1.00]	**0.055**	3151	0.79 [0.58,1.08]	0.138
Boys	1834	0.86 [0.65,1.15]	0.315	1582	0.97 [0.64,1.46]	0.881
Girls	1753	0.72 [0.51,1.03]	**0.072**	1569	0.63 [0.39,0.997]	**0.049**
Mother smokes						
All children	462	0.84 [0.44,1.59]	0.597	365	0.87 [0.30,2.51]	0.790
Boys	232	0.59 [0.25,1.38]	0.220	178	0.34 [0.07,1.66]	0.183
Girls	230	1.49 [0.53,4.20]	0.454	187	9.75 [0.72,133]	**0.087**
					*	

*Significant difference between results for boys and girls; AOR = Adjusted odds ratio

#### Grandchild’s setting of music listening level

The test of music listening level was repeated on two occasions (named run 1 and run 2). In order to determine whether choice of run made any difference to the results we compared the two sets of unadjusted mean results. They showed the same associations–we therefore decided to use Run 1 for further analyses since there were marginally more results for this than for Run 2 (S3A and S3B Table). The comparisons showed that the grandchildren whose grandmothers had smoked when expecting either parent tended to set their volume level higher than those whose grandmothers had not smoked in pregnancy, and that the granddaughters set their level higher than the grandsons.

In order to compare our results with those who were intolerant loud noise, we have analysed the volume listening results assuming that those who were intolerant of loud sounds would be less likely to choose the highest 10% volume levels. The unadjusted results for maternal grandmothers smoking in pregnancy (Table 6) indicate that the grandchildren, especially the granddaughters, were more likely to choose a louder volume. As before, we assessed the backgrounds of the grandparents to identify what variables to allow for (S4 Table).

**Table 6:** Odds ratio showing risk of stereo run 1 volume level being in the top 10% where maternal grandmother smoked. Adjustment A concerns allowing for maternal grandmother’s year of birth, her ethnicity, her education level; Adjustment B additionally takes the maternal grandfather’s social class into account.

Stereo Run 1 in top 10%	Unadjusted			Adjustment A			Adjustment B		
	N	UOR [95%CI]	P	N	AOR [95% CI]	P	N	AOR [95% CI]	P
All children	3950	1.34 [1.08,1.66]	**0.008**	2709	1.13 [0.86,1.50]	0.382	2323	1.02 [0.75,1.39]	0.906
Boys	1972	1.09 [0.82,1.44]	0.552	1359	0.88 [0.61,1.27]	0.490	1171	0.72 [0.48,1.08]	0.115
Girls	1978	1.92 [1.36,2.72]	**0.0002**	1350	1.71 [1.09,2.69]	**0.020**	1152	1.75 [1.07,2.88]	**0.027**
		*			*			*	
Mother non-smoker									
All children	3386	1.28 [1.00,1.62]	**0.046**	2393	1.08 [0.79,1.48]	0.623	2068	0.92 [0.65,1.30]	0.651
Boys	1688	0.99 [0.72,1.36]	0.957	1196	0.80 [0.53,1.21]	0.296	1036	0.63 [0.40,0.99]	**0.049**
Girls	1698	1.96 [1.34,2.88]	**0.001**	1197	1.74 [1.05,2.88]	**0.031**	1197	1.64 [0.95,2.83]	**0.078**
		*			*			*	
Mother smoked									
All children	551	1.44 [0.86,2.38]	0.162	310	1.13 [0.57,2.21]	0.727	250	1.53 [0.70,3.34]	0.283
Boys	280	1.47 [0.77,2.81]	0.241	162	1.13 [0.48,2.66]	0.776	134	1.25 [0.46,3.36]	0.665
Girls	271	1.43 [0.62,3.27]	0.401	148	1.11 [0.36,3.45]	0.859	116	2.05 [0.55,7.72]	0.287

*Significant difference between results for boys and girls; UOR = unadjusted odds ratio; AOR = adjusted odds ratio

The factors that were relevant for the maternal line were the maternal grandmother’s year of birth, her ethnicity, her education level and the maternal grandfather’s social class. The relationships without and then including social class are shown in Table 6. Whether adjusted or not, there were significant interactions between the sexes–granddaughters whose mother had been exposed as a fetus to their grandmother smoking were significantly more likely to choose loud sounds than the grandsons, who tended to be less likely to make this choice. This was true of the population as a whole as well as of those grandchildren whose mothers did not smoke. If there was any association among grandchildren whose mothers smoked in pregnancy, the numbers of children involved were too small to demonstrate this.

For the paternal line, the factors adjusted for were the paternal grandfather’s year of birth, his highest educational qualification, and his social class. Although the results showed few unadjusted associations, again there were significant interactions, with granddaughters more likely to choose loud sounds and boys less so (Table 7).

**Table 7:** Odds ratio showing risk of stereo run 1 volume level being in the top 10% where paternal grandmother smoked. Adjustment A concerns allowing for paternal grandmother’s year of birth, her ethnicity, and her education level; Adjustment B additionally takes the paternal grandfather’s social class into account.

Stereo Run 1 in top 10%	Unadjusted			Adjustment A			Adjustment B		
	N	UOR [95%CI]	P	N	AOR [95% CI]	P	N	AOR [95% CI]	P
All children	3185	1.23 [0.97,1.57]	**0.085**	1644	1.10 [0.77,1.58]	0.609	1560	1.08 [0.74,1.57]	0.691
Boys	1600	1.12 [0.83,1.50]	0.469	828	0.85 [0.53,1.34]	0.481	777	0.86 [0.53,1.38]	0.531
Girls	1585	1.57 [1.04,2.37]	**0.033**	816	1.73 [0.94,3.17]	**0.077**	783	1.59 [0.84,2.99]	0.151
					*			*	
Mother non-smoker									
All children	2786	1.14 [0.88,1.49]	0.327	1490	0.98 [0.66,1.45]	0.914	1415	0.95 [0.63,1.43]	0.802
Boys	1400	1.02 [0.73,1.42]	0.911	756	0.77 [0.47,1.28]	0.313	710	0.78 [0.46,1.30]	0.336
Girls	1386	1.45 [0.92,2.29]	0.105	734	1.45 [0.75,2.79]	0.266	705	1.34 [0.67,2.67]	0.409
					*			*	
Mother smoked									
All children	384	1.40 [0.78,2.52]	0.261	147	1.95 [0.66,5.76]	0.227	139	2.20 [0.67,7.18]	0.192
Boys	193	1.30 [0.64,2.68]	0.470	70	1.00 [0.24,4.24]	0.997	66	1.02 [0.20,5.13]	0.976
Girls	191	1.98 [0.66, 5.95]	0.221	77	6.25 [0.67,58.0]	0.107	73	6.83 [0.68,68.9]	0.103

*Significant difference between results for boys and girls; UOR = unadjusted odds ratio; AOR = adjusted odds ratio

### Grandchild’s hearing function

There were no significant associations between either maternal or paternal grandmother’s smoking and auditory reflex threshold, hearing threshold, tinnitus or whether the child had ever been referred for hearing loss (S4–S6 Tables).

## Discussion

Our aim was to assess whether the grandchild’s intolerance to loud sounds had an intergenerational association with the grandmother’s smoking during the pregnancy that had resulted in the birth of the grandchild’s parent. We compared the grandchildren whose grandmothers smoked during pregnancy with the grandchildren whose grandmothers did not, analysing separately the maternal and paternal grandmothers. As in our previous studies **[6, 21, 22]**, we considered the population as a whole as well as subdividing by sex of the grandchild and by whether the grandchild’s mother had smoked or not during the pregnancy.

We showed that about 11% of the 6-year-old grandchildren were said to be intolerant to loud sound. If the maternal grandmother had smoked in pregnancy the boys were more likely than expected, and the girls were less likely than expected to be so (P for interaction <0.05). This pattern was found for all grandchildren considered together, as well as for those whose mothers did not themselves smoke in pregnancy. There were no such relationships for 13-year-old grandchildren (Tables 1 and 3).

For paternal grandmothers’ smoking, the grandchildren tended to be less likely than expected to be intolerant of loud noises at both age 6 and 13, but the only interaction between the sexes was in the relatively small group comprising both mother and paternal grandmother smoking prenatally (the 13-year-old grandsons were less likely and the granddaughters more likely to hate loud sounds (Tables 4 and 5)). However, with much larger numbers, the granddaughters of grandmothers who had and mothers who had not smoked prenatally were significantly less likely to have such a reaction to loud noises.

Since the report of intolerance to loud sounds was based on subjective reports from parents, we used the preferred volume listening level set by the child themselves as a more objective measure. We assumed that children who were intolerant to loud sounds would be less likely to choose the loudest part of the register. As predicted, we found that, if the grandmother had smoked in pregnancy, not only were the grandsons more likely than the granddaughters to be said to be intolerant to loud sounds; confirmatory evidence was shown on testing–the grandsons were less likely than the granddaughters to choose to listen at higher volume levels (Tables 6 and 7).

There was no association of peripheral auditory function with grandmother’s smoking, indicating that the observed effect is not likely to be explained by differences in hearing thresholds. The question about sound intolerance asked in this study specifically relates to loud sounds, rather than intolerance to everyday or low intensity sounds (known as hyperacusis). Intolerance to loud sounds may relate to the phenomenon of loudness recruitment, a characteristic of cochlear hearing loss **[60]**. However, the lack of an association with hearing threshold suggests this is not a peripheral, cochlear phenomenon. The findings are consistent with models of hyperacusis which propose a central rather than peripheral cause, resulting from increased central auditory gain **[61]**. However as noted above, the measure used in this paper is not synonymous with hyperacusis **[62]**.

At present we are largely ignorant of the causal pathways underpinning this and similar intergenerational responses to grandmaternal smoking in pregnancy. As indicated in the Introduction, the exposure of either (F1) parent to tobacco as a fetus may have resulted in a direct xenobiotic exposure to both their developing somatic tissues and their emerging germline, ultimately destined for any (F2) grandchildren. Alternatively, it may be the generalised DNA damage caused by (FO) grandmaternal smoking and/or the consequent DNA damage response of the fetus that modifies the emerging germline and alters F2 embryonic brain development. Clearly very complex, it is premature to speculate further.

Explaining the sex differences observed here, and in those reported in many earlier studies of inter/trans-generational responses, might seem more tractable. However, a recent lengthy review of the role of sex in the genomics of human complex traits emphasises that this has been a neglected topic **[63]**. These authors note that nearly all human complex traits and disease phenotypes exhibit some degree of sex differences, but attempts to attribute this solely to classic genetic x environmental effects has proved unfruitful. Despite devoting some space to epigenome analysis and genomic imprinting, the authors only reviewed single generation studies and assumed throughout that biological inheritance is purely down to transmission of DNA sequence differences. A meta-analysis of 2,335,920 twin pairs and 2,608 phenotypes reports that only 3% demonstrated significant sex differences in heritability **[64]**. Furthermore, classic twin studies are likely to be confounded by epigenetic super-similarity in monozygotic twins **[43]**. One conclusion from the failure of these huge studies to explain little of the sex biases in complex diseases and traits is that non-DNA sequence-based inheritance may be an important source of sex differences in developmental traits.

### Strengths and limitations

The strengths of this study concern: (a) the fact that it is population based with a large number of subjects; (b) the fact that data on the grandparents were collected during pregnancy, long before the child’s sensitivity to loud sound could be identified; (c) the analysis was structured around the hypothesis that, like previous intergenerational associations with grandparental prenatal smoking, any associations observed in the grandchildren would show sex differences; (d) the outcome measures used were able to be verified with an objective measure (the volume which the grandchild set the CD player).

Limitations concern the fact that information on the child’s intolerance to loud sound are answered by the mother, and consequently are likely to depend on her own observational skills, and the general noise level in the home. However, this criticism is not valid for the direct measures of sound volume preferences using the headphones. A further limitation concerns the fact that the smoking history of the grandmother was collected retrospectively; nevertheless comparison of the birthweights of the daughters of the grandmothers reported as smoking prenatally with those who were not indicates that these data have validity. The major limitation of the study concerns the fact that, at this point in time, there is no evidence (confirmatory or otherwise) from other studies.

## Supporting information

S1 Table The proportion of study pregnancies that resulted in a child for whom the questions on sound intolerance were answered at ages 6 and 13 together with the proportion for whom the stereo test results were available according to whether the parent was born to a grandmother who smoked during their pregnancy. (DOCX)

S2 Table Proportion (n) of children who hated loud noises at age 13 according to features of their grandparents. [P values <0.10 are in bold].
(DOCX)

S3 Table a. Unadjusted mean stereo by maternal grandmother smoked. b. Unadjusted mean stereo by paternal grandmother smoked. [P values <0.10 are in bold].
(DOCX)

S4 Table Highest 10% of stereo level chosen by the children at age 11 according to features of their grandparents. [P values <0.10 are in bold].
(DOCX)

S5 Table Risk of the child having a flat acoustic reflex at age 11 if the maternal grandmother had smoked in pregnancy. [P values <0.10 are in bold].
(DOCX)

S6 Table The prevalences of hearing features of the children at age 11 according to whether a grandmother had smoked prenatally when expecting the study parent. (DOCX)
