# Supplementary material for: Intolerance of loud sounds in childhood: Is there an intergenerational association with grandmaternal smoking in pregnancy?
Source: PLoS One. 2020 Feb 24;15(2):e0229323. doi: 10.1371/journal.pone.0229323 (PMC7039668; doi:10.1371/journal.pone.0229323)
Supplement: S1 Table — (DOCX) [file pone.0229323.s001.docx]

S1 Table. The proportion of study pregnancies that resulted in a child for whom the questions on sound intolerance were answered at ages 6 and 13 together with the proportion for whom the stereo test results were available according to whether the parent was born to a grandmother who smoked during their pregnancy.

| **OUTCOME** | **MGM** | **MGM** |  | **PGM** | **PGM** |
| --- | --- | --- | --- | --- | --- |
| **AND AGE** | **SMOKED** | **DID NOT SMOKE** |  | **SMOKED** | **DID NOT SMOKE** |
|  |  |  |  |  |  |
| Hates loud noise | 57.4% (2749) | 60.8% (5124) |  | 64.2% (2597) | 68.6% (3815) |
| 6 years |  |  |  |  |  |
|  |  |  |  |  |  |
| Hates loud noise | 45.6% (2185) | 49.8% (4198) |  | 51.6% (2087) | 57.4% (3192) |
| 13 years |  |  |  |  |  |
|  |  |  |  |  |  |
| Stereo test | 27.8% (1330) | 31.1% (2620) |  | 31.5% (1272) | 34.4% (1914) |
| 11 years |  |  |  |  |  |
|  |  |  |  |  |  |
|  |  |  |  |  |  |

MGM = maternal grandmother; PGM = paternal grandmother
