# Supplementary material for: Intolerance of loud sounds in childhood: Is there an intergenerational association with grandmaternal smoking in pregnancy?
Source: PLoS One. 2020 Feb 24;15(2):e0229323. doi: 10.1371/journal.pone.0229323 (PMC7039668; doi:10.1371/journal.pone.0229323)
Supplement: S2 Table — [P values <0.10 are in bold]. (DOCX) [file pone.0229323.s002.docx]

S2 Table. Proportion (n) of children who hated loud noises at age 13 according to features of their grandparents. [P values <0.10 are in bold].

| **Variable** | **MGM** | **MGF** | **PGM** | **PGF** |
| --- | --- | --- | --- | --- |
|  |  |  |  |  |
| Year of birth |  |  |  |  |
| Pre 1925 | 6.66% (42) | 6.52% (70) | 8.04% (53) | 7.32% (75) |
| 1925-1929 | 6.86% (50) | 7.05% (73) | 5.85% (39) | 6.79% (51) |
| 1930-1934 | 5.54% (71) | 5.28% (68) | 5.92% (52) | 5.70% (44) |
| 1935-1939 | 5.44% (77) | 5.09% (61) | 6.39% (47) | 4.93% (30) |
| 1940-1944 | 5.00% (56) | 4.64% (35) | 5.76% (31) | 4.89% (15) |
| 1945+ | 4.98%(38) | 5.34% (21) | 1.94% (5) | 2.48% (3) |
| P | **0.039** | **0.023** | **0.009** | **0.003** |
| N | 6069 | 5742 | 3737 | 3604 |
|  |  |  |  |  |
| Ethnic background | |  |  |  |
| White | 5.53% (356) | 5.51% (353) | 5.56% (290) | 5.55% (288) |
| Non-white | 9.38% (9) | 9.35% (10) | 6.82% (6) | 6.96% (8) |
| P | 0.108 | **0.091** | 0.608 | 0.518 |
| N | 6532 | 6512 | 5312 | 5301 |
|  |  |  |  |  |
| Education level |  |  |  |  |
| Lower | 5.78% (181) | 5.89% (165) | 5.59% (150) | 5.93% (145) |
| Higher | 6.05% (116) | 5.83% (115) | 6.00% (88) | 5.70% (97) |
| P | 0.683 | 0.923 | 0.589 | 0.750 |
| N | 5050 | 4774 | 4147 | 4149 |
|  |  |  |  |  |
| Ever smoked |  |  |  |  |
| Yes | 5.24% (179) | 5.26% (248) | 5.16% (150) | 5.50% (212) |
| No | 5.85% (175) | 6.16% (100) | 6.23% (149) | 6.54% (71) |
| P | 0.279 | 0.171 | **0.093** | 0.192 |
| N | 6408 | 6339 | 5302 | 4944 |
|  |  |  |  |  |
| Age at birth of parent | |  |  |  |
| <25 years | 5.64% (121) | 5.17% (55) | 4.73% (72) | 4.01% (31) |
| 25-34 | 5.46% (174) | 5.64% (189) | 5.91% (146) | 5.64% (149) |
| 35+ | 6.54% (48) | 6.33% (84) | 7.89% (49) | 6.68% (75) |
| P | 0.557 | 0.177 | **0.005** | **0.049** |
| N | 6069 | 5742 | 4615 | 4437 |
|  |  |  |  |  |
| Parity |  |  |  |  |
| 0 | 5.45% (116) | - | 6.70% (55) | - |
| 1+ | 5.62% (252) |  | 5.26% (68) |  |
| P | 0.778 |  | 0.157 |  |
| N | 6611 |  | 2110 |  |
|  |  |  |  |  |
| Smoked prenatally | |  |  |  |
| Yes | 5.26% (115) | - | 4.65% (97) | - |
| No | 5.65% (237) |  | 6.30% (201) |  |
| P | 0.525 |  | **0.011** |  |
| N | 6383 |  | 5279 |  |
|  |  |  |  |  |
| Social class |  |  |  |  |
| P | 0.009 | 0.370 | 0.664 | 0.206 |
| N | 3664 | 5452 | 2819 | 4934 |
