# Supplementary material for: Intolerance of loud sounds in childhood: Is there an intergenerational association with grandmaternal smoking in pregnancy?
Source: PLoS One. 2020 Feb 24;15(2):e0229323. doi: 10.1371/journal.pone.0229323 (PMC7039668; doi:10.1371/journal.pone.0229323)
Supplement: S3 Table — a. Unadjusted mean stereo by maternal grandmother smoked. b. Unadjusted mean stereo by paternal grandmother smoked. [P values <0.10 are in bold]. (DOCX) [file pone.0229323.s003.docx]

S3a Table. Unadjusted mean stereo by **maternal** grandmother smoked. [P values <0.10 are in bold].

| **HATES LOUD SOUNDS** | **Run 1** | | | **Run 2** | | |
| --- | --- | --- | --- | --- | --- | --- |
|  | **N** | **MD [95%CI]** | **P** | **N** | **MD [95% CI]** | **P** |
|  |  |  |  |  |  |  |
| All children | 3956 | +0.59 [+0.20,+0.98] | **0.003** | 3952 | +0.63 [+0.24, +1.02] | **0.002** |
| Boys | 1976 | +0.24 [-0.35,+0.83] | 0.422 | 1974 | +0.27 [-0.32, +0.86] | 0.370 |
| Girls | 1980 | +0.99 [+0.48,+1.49] | **<0.001** | 1978 | +1.02 [+0.52, +1.52] | **<0.0001** |
|  |  | * |  |  | * |  |
| Mother non-smoker | | |  |  |  |  |
| All children | 3392 | +0.48 [+0.06,+0.91] | **0.026** | 3388 | +0.55 [+0.12, +0.97] | **0.011** |
| Boys | 1692 | +0.15 [-0.49,+0.79] | 0.644 | 1690 | +0.19 [-0.45, +0.83] | 0.559 |
| Girls | 1700 | +0.86 [+0.32,+1.41] | **0.002** | 1698 | +0.95 [+0.41, +1.49] | **<0.001** |
|  |  | * |  |  | * |  |
| Mother smokes | |  |  |  |  |  |
| All children | 551 | +0.71 [-0.32,+1.74] | 0.176 | 551 | +0.57 [-0.47, +1.61] | 0.283 |
| Boys | 280 | +0.54 [-1.00,+2.08] | 0.489 | 280 | +0.52 [-1.04, +2.09] | 0.510 |
| Girls | 271 | +0.91 [-0.46,+2.28] | 0.193 | 271 | +0.64 [-0.74, +2.01] | 0.362 |
|  |  |  |  |  |  |  |

*Significant difference between results for boys and girls; MD = mean difference

S3b Table. Unadjusted mean stereo by **paternal** grandmother smoked. [P values <0.10 are in bold].

| **HATES LOUD SOUNDS** | **Run 1** | | | **Run 2** | | |
| --- | --- | --- | --- | --- | --- | --- |
|  | **N** | **MD [95%CI]** | **P** | **N** | **MD [95% CI]** | **P** |
|  |  |  |  |  |  |  |
| All children | 3189 | +0.48 [+0.06,+0.89] | **0.024** | 3187 | +0.44 [+0.03,+0.85] | **0.036** |
| Boys | 1603 | +0.37 [-0.25,+1.00] | 0.243 | 1602 | +0.28 [-0.35,+0.91] | 0.381 |
| Girls | 1586 | +0.63 [+0.11,+1.15] | **0.019** | 1585 | +0.65 [+0.14,+1.17] | **0.013** |
|  |  |  |  |  |  |  |
| Mother non-smoker | | |  |  |  |  |
| All children | 2792 | +0.36 [-0.08,+0.80] | 0.109 | 2790 | +0.35 [-0.09,+0.79] | 0.116 |
| Boys | 1404 | +0.29 [-0.38,+0.95] | 0.396 | 1403 | +0.23 [-0.44,+0.89] | 0.502 |
| Girls | 1388 | +0.46 [-0.10,+1.01] | 0.105 | 1387 | +0.50 [-0.04,+1.05] | **0.072** |
|  |  |  |  |  |  |  |
| Mother smokes | |  |  |  |  |  |
| All children | 384 | +0.74 [-0.53,+2.01] | 0.255 | 384 | +0.62 [-0.66,+1.91] | 0.341 |
| Boys | 193 | +0.35 [-1.60,+2.31] | 0.723 | 193 | +0.15 [-1.84,+2.14] | 0.884 |
| Girls | 191 | +1.34 [-0.26,+2.95] | 0.101 | 191 | +1.30 [-0.30,+2.90] | 0.111 |
|  |  |  |  |  |  |  |

*Significant difference between results for boys and girls

MD = mean difference
