# Supplementary material for: Intolerance of loud sounds in childhood: Is there an intergenerational association with grandmaternal smoking in pregnancy?
Source: PLoS One. 2020 Feb 24;15(2):e0229323. doi: 10.1371/journal.pone.0229323 (PMC7039668; doi:10.1371/journal.pone.0229323)
Supplement: S4 Table — [P values <0.10 are in bold]. (DOCX) [file pone.0229323.s004.docx]

S4 Table. Highest 10% of stereo level chosen by the children at age 11 according to features of their grandparents. [P values <0.10 are in bold].

| **Variable** | **MGM** | **MGF** | **PGM** | **PGF** |
| --- | --- | --- | --- | --- |
|  |  |  |  |  |
| Year of birth |  |  |  |  |
| Pre 1925 | 9.3% (35) | 9.9% (63) | 8.0% (31) | 6.6% (38) |
| 1925-1929 | 9.9% (49) | 9.8% (58) | 8.0% (29) | 8.4% (35) |
| 1930-1934 | 9.6% (75) | 7.5% (59) | 7.0% (36) | 9.5% (44) |
| 1935-1939 | 8.7% (74) | 10.4% (80) | 10.6% (46) | 10.7% (40) |
| 1940-1944 | 9.9% (74) | 8.9% (43) | 10.2% (35) | 9.2% (17) |
| 1945+ | 13.7% (69) | 17.8% (49) | 10.5% (15) | 11.3% (8) |
| P | **0.081** | **0.041** | **0.095** | **0.024** |
| N | 3760 | 3546 | 2186 | 2091 |
|  |  |  |  |  |
| Ethnic background | |  |  |  |
| White | 10.1% (398) | 10.0% (395) | 9.6% (302) | 9.5% (298) |
| Non-white | 18.0% (11) | 15.7% (11) | 15.1% (8) | 15.2% (10) |
| P | **0.045** | 0.123 | 0.181 | 0.128 |
| N | 4017 | 4004 | 3211 | 3202 |
|  |  |  |  |  |
| Education level |  |  |  |  |
| Lower | 11.0% (210) | 11.2% (195) | 10.0% (164) | 10.8% (165) |
| Higher | 7.4% (85) | 7.8% (89) | 8.1% (69) | 7.4% (73) |
| P | **0.001** | **0.003** | 0.108 | **0.005** |
| N | 3064 | 2876 | 2490 | 2505 |
|  |  |  |  |  |
| Ever smoked |  |  |  |  |
| Yes | 10.7% (227) | 10.1% (293) | 10.5% (186) | 9.9% (233) |
| No | 9.4% (166) | 9.4% (96) | 8.4% (120) | 8.0% (52) |
| P | **0.062** | 0.507 | **0.045** | 0.142 |
| N | 3967 | 3926 | 3199 | 2992 |
|  |  |  |  |  |
| Age at birth of parent | |  |  |  |
| <25 years | 10.8% (147) | 12.1% (82) | 10.6% (92) | 11.0% (49) |
| 25-34 | 9.0% (176) | 8.6% (179) | 7.9% (119) | 8.3% (126) |
| 35+ | 11.7% (53) | 11.3% (54) | 11.6% (45) | 9.6% (64) |
| P | 0.796 | **0.027** | **0.024** | 0.595 |
| N | 3760 | 3546 | 2761 | 2639 |
|  |  |  |  |  |
| Parity |  |  |  |  |
| 0 | 9.7% (126) | - | 9.1% (43) | - |
| 1+ | 10.5% (289) |  | 9.1% (68) |  |
| P | 0.447 |  | 0.935 |  |
| N | 4064 |  | 1235 |  |
|  |  |  |  |  |
| Smoked prenatally | |  |  |  |
| Yes | 11.7% (155) | - | 10.6% (135) | - |
| No | 9.0% (235) |  | 8.8% (168) |  |
| P | **0.008** |  | **0.085** |  |
| N | 3950 |  | 3185 |  |
|  |  |  |  |  |
| Social class |  |  |  |  |
| P | **0.028** | **<0.0001** | **0.070** | **0.002** |
| N | 2292 | 3368 | 1663 | 2962 |
