# Supplementary material for: Intolerance of loud sounds in childhood: Is there an intergenerational association with grandmaternal smoking in pregnancy?
Source: PLoS One. 2020 Feb 24;15(2):e0229323. doi: 10.1371/journal.pone.0229323 (PMC7039668; doi:10.1371/journal.pone.0229323)
Supplement: S5 Table — [P values <0.10 are in bold]. (DOCX) [file pone.0229323.s005.docx]

S5 Table. Risk of the child having a flat acoustic reflex at age 11 if the **maternal** grandmother had smoked in pregnancy. [P values <0.10 are in bold].

|  | **LEFT EAR** | | | **RIGHT EAR** | | |
| --- | --- | --- | --- | --- | --- | --- |
|  | **N** | **UOR [95%CI]** | **P** | **N** | **UOR [95% CI]** | **P** |
|  |  |  |  |  |  |  |
| All children | 5530 | 0.98 [0.82,1.16] | 0.784 | 5212 | 1.03 [0.87,1.23] | 0.734 |
| Boys | 2662 | 1.12 [0.88,1.43] | 0.370 | 2463 | 0.96 [0.74,1.26] | 0.788 |
| Girls | 2868 | 0.85 [0.66,1.09] | 0.202 | 2749 | 1.08 [0.86,1.36] | 0.500 |
|  |  | * |  |  |  |  |
| Mother non-smoker | | |  |  |  |  |
| All children | 4769 | 0.96 [0.79,1.16] | 0.637 | 4471 | 1.04 [0.86,1.26] | 0.674 |
| Boys | 2306 | 1.06 [0.81,1.39] | 0.649 | 2120 | 0.97 [0.72,1.29] | 0.822 |
| Girls | 2463 | 0.85 [0.65,1.12] | 0.262 | 2351 | 1.10 [0.85,1.42] | 0.458 |
|  |  |  |  |  |  |  |
| Mother smoker | |  |  |  |  |  |
| All children | 741 | 1.19 [0.75,1.88] | 0.459 | 721 | 0.93 [0.61,1.42] | 0.746 |
| Boys | 352 | 1.81 [0.90,3.64] | **0.098** | 339 | 0.89 [0.47,1.70] | 0.733 |
| Girls | 389 | 0.85 [0.45,1.59] | 0.613 | 382 | 0.97 [0.55, 1.69] | 0.905 |
|  |  | * |  |  |  |  |

*Significant difference between results for boys and girls; UOR = unadjusted odds ratio

UOR = unadjusted odds ratio
