# Supplementary material for: Intolerance of loud sounds in childhood: Is there an intergenerational association with grandmaternal smoking in pregnancy?
Source: PLoS One. 2020 Feb 24;15(2):e0229323. doi: 10.1371/journal.pone.0229323 (PMC7039668; doi:10.1371/journal.pone.0229323)
Supplement: S6 Table — (DOCX) [file pone.0229323.s006.docx]

S6 Table. The prevalences of hearing features of the children at age 11 according to whether a grandmother had smoked prenatally when expecting the study parent.

| **MEASURE** | **MGM+** | **MGM-** | **P** | **PGM+** | **PGM-** | **P** |
| --- | --- | --- | --- | --- | --- | --- |
|  |  |  |  |  |  |  |
|  | **% (n)** | **% (n)** |  | **% (n)** | **% (n)** |  |
|  |  |  |  |  |  |  |
| Tinnitus in both ears | 17.2 (362) | 15.5 (650) | 0.094 | 15.9 (327) | 16.4 (503) | 0.616 |
|  |  |  |  |  |  |  |
| Acoustic reflex flat-  Right ear | 12.6 (221) | 12.3 (424) | 0.734 | 11.7 (200) | 12.7 (321) | 0.344 |
| Acoustic reflex flat-  Left ear | 11.4 (214) | 11.7 (428) | 0.784 | 10.8 (196) | 11.7 (315) |  |
| Referred for suspected hearing loss ever by age 11 | 20.5 (434) | 18.9 (794) | 0.138 | 19.8 (409) | 19.1 (590) | 0.571 |
|  |  |  |  |  |  |  |
|  | *Mean (SD)* | *Mean (SD)* |  | *Mean (SD)* | *Mean (SD)* |  |
| Average of 0.5, 1, 2, 4kHz |  |  |  |  |  |  |
| Right ear | 4.06 (6.62) | 3.93 (6.24) | 0.467 | 4.06 (6.37) | 3.83 (6.30) | 0.203 |
| Left ear | 4.02 (6.73) | 4.05 (6.52) | 0.901 | 4.04 (6.51) | 3.87 (6.41) | 0.343 |
